# Does hemipelvis structure and position influence acetabulum orientation?

**DOI:** 10.1186/s12891-016-0982-2

**Published:** 2016-03-16

**Authors:** Bartosz Musielak, Marek Jóźwiak, Michał Rychlik, Brian Po-Jung Chen, Maciej Idzior, Andrzej Grzegorzewski

**Affiliations:** Department of Paediatric Orthopaedics and Traumatology, Poznan University of Medical Sciences, Fredry 10, Poznan, 61-701 Poland; Division of Virtual Engineering, Poznan University of Technology, Piotrowo 3, Poznan, 60-965 Poland; Department of Orthopaedics and Paediatric Orthopaedics, Medical University of Lodz, Kościuszki 4, Łódź, Poland

**Keywords:** Pelvic bone, Hip joint, Spatial orientation, Computed tomography, Sacral base plane

## Abstract

**Background:**

Although acetabulum orientation is well established anatomically and radiographically, its relation to the innominate bone has rarely been addressed. If explored, it could open the discussion on patomechanisms of such complex disorders as femoroacetabular impingement (FAI). We therefore evaluated the influence of pelvic bone position and structure on acetabular spatial orientation. We describe this relation and its clinical implications.

**Methods:**

This retrospective study was based on computed tomography scanning of three-dimensional models of 31 consecutive male pelvises (62 acetabulums). All measurements were based on CT spatial reconstruction with the use of highly specialized software (Rhinoceros). Relations between acetabular orientation (inclination, tilt, anteversion angles) and pelvic structure were evaluated. The following parameters were evaluated to assess the pelvic structure: iliac opening angle, iliac tilt angle, interspinous distance (ISD), intertuberous distance (ITD), height of the pelvis (HP), and the ISD/ITD/HP ratio. The linear and nonlinear dependence of the acetabular angles and hemipelvic measurements were examined with Pearson’s product − moment correlation and Spearman’s rank correlation coefficient. Correlations different from 0 with *p* < 0.05 were considered statistically significant.

**Results:**

Comparison of the axis position with pelvis structure with orientation in the horizontal plane revealed a significant positive correlation between the acetabular anteversion angle and the iliac opening angle (*p* = 0.041 and 0.008, respectively). In the frontal plane, there was a positive correlation between the acetabular inclination angle and the iliac tilt angle (*p* = 0.025 and 0.014, respectively) and the acetabular inclination angle and the ISD/ITD/HP ratio (both *p* = 0.048).

**Conclusions:**

There is a significant correlation of the hemipelvic structure and acetabular orientation under anatomic conditions, especially in the frontal and horizontal planes. In the anteroposterior view, the more tilted-down innominate bone causes a more caudally oriented acetabulum axis, whereas in the horizontal view this relation is reversed. This study may serve as a basis for the discussion on the role of the pelvis in common disorders of the hip.

## Background

Acetabular orientation based on anatomic specimens is well known physiologically and pathologically [1–4]. Radiographic knowledge of orientation is increasing rapidly, not only based on two-dimensional (2D) imaging but strongly related to the three-dimensional (3D) examinations, with ongoing discussion on the subject of reference planes [5–11]. The orientation of the hip socket in these situations is interpreted in relation to basic reference planes. The importance of acetabular direction (more precisely its retroversion) on development of hip pathologies is evident in diseases such as neurogenic hip disease [12, 13]. In the literature, however, the topic of the relation of the acetabulum to the innominate bone is rarely addressed. Pathology of the entire innominate bone, not only the acetabulum, in typical hip joint disorders is often ignored. The only described acetabular pathology of the hemipelvis has been in regard to developmental dysplasia of the hip (DDH) [14–16]. Some authors proposed that growth disturbances affect not only the acetabulum but also the entire innominate bone [14, 17, 18]. To our knowledge, no one has focused on the influence of innominate bone position in one of the major conditions leading to osteoarthritis of the hip, femoroacetabular impingement, (FAI), in which the influence of the position of the entire pelvis on conflict of the femur and acetabulum has been reported several times [19, 20]. There have also been no studies that assessed the influence of the innominate bone position on the acetabulum under physiologic conditions. This limits our understanding of hip physiology and thus that of the pathogenesis of various hip pathologies, such as DDH and impingement syndrome, where it would be important to know whether and how the entire pelvis is affected [14].

Thus, the aim of our research was to evaluate the influence of the pelvic bone position and structure on acetabular spatial orientation based on computed tomography (CT) evaluation. We also evaluated the existing relations and their clinical implications.

## Methods

**Fig. 1:**
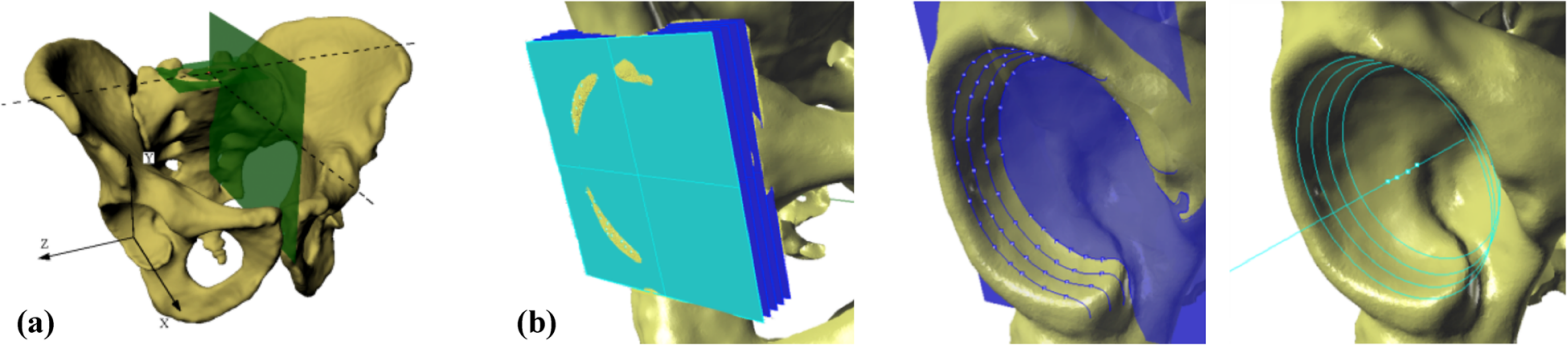
**a** Sacrum Base (SB) plane with corresponding saggital plane and the coordinate system XYZ, **b** The acetabulum axis set on the 3D model

**Fig. 2:**
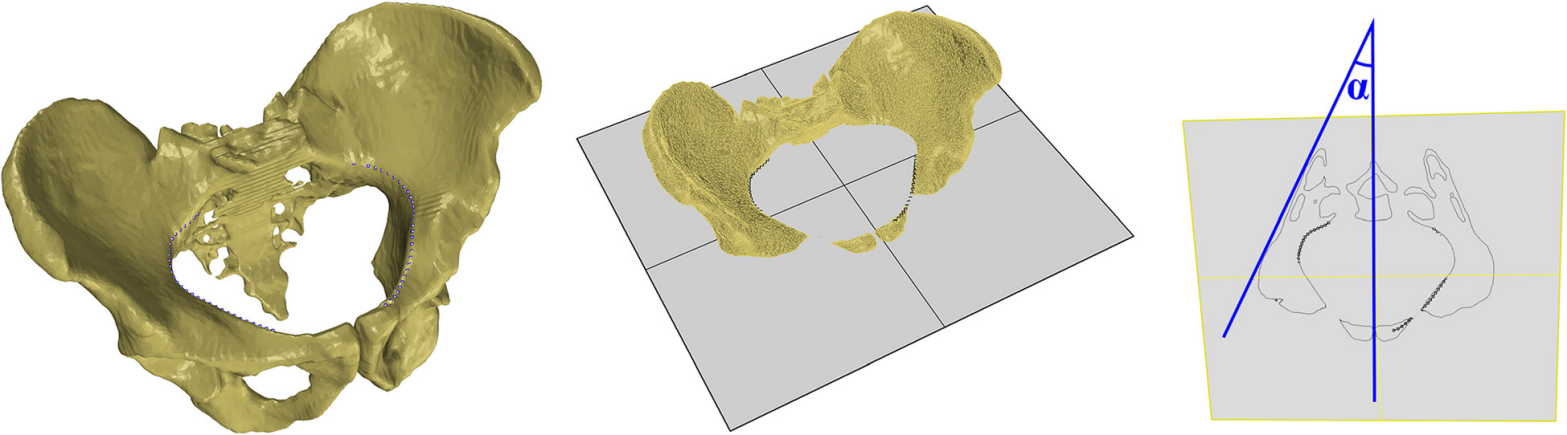
Iliac opening angle - measured in the inlet plane cross-section with process of its generation from the 3D model (α angle - representing right Iliac opening angle, measured between the line tangential to the posterolateral wall of the ischial bone and the sagittal axis

**Fig. 3:**
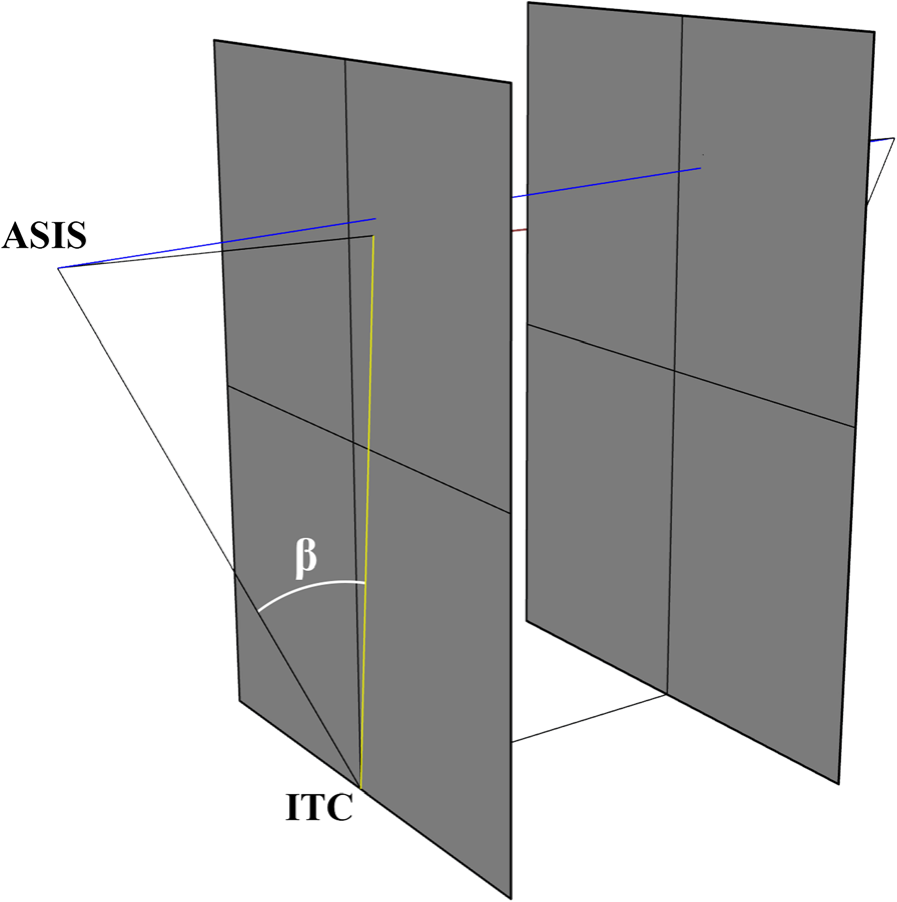
Iliac tilt angle - measured in true a-p plane, based on the planes acquired from the 3D model

This retrospective study utilized 3D models of CT scans of 31 consecutive adult male pelvices from patients who were examined because of surgical, not orthopedic, diagnoses during 2012–2014. None of the patients included in this study had pelvic bone lesions. Only scans containing the whole bony pelvis were considered acceptable (62 acetabulums and hemipelves). The CT scans used in this study were attained with the use of GE LightSpeed VCT 64 Slice CT (GE Healthcare Milwaukee, WI, USA). The slice thickness of the accepted scans was 0.63 or 1.25 mm. Scans formatted as digital imaging and communication in medicine (DICOM) files were transferred to computer-assisted diagnostic software for comprehensive processing of the 3D image data (ScanIP, Simpleware, Exeter, UK) and subsequently for measurements on spatial images based on the obtained 3D model (Rhinoceros, Robert McNeel & Associates, Seattle, WA, USA; ScanIP, Simpleware, Exeter, UK). As a reference plane for further measurement, we used the sacral base plane (Fig. 1a), which was determined as the plane interpolated from the mesh of points (over 100), which were applied on the surface of the sacrum base. This plane was described and thoroughly discussed previously by Jozwiak et al. [10]. We set the axis of the acetabulum on the 3D model using the acetabular opening plane and centers of circles interpolated on planes parallel to the acetabular opening, cutting a hip socket into 1-mm slices (Fig. 1b). This method was also described previously by Jozwiak et al. [10]. We then measured the innominate bone dimensions and orientations on the 3D pelvic model, including the iliac opening angle measured in the inlet plane (Fig. 2), the iliac tilt angle measured in the true anteroposterior (AP) plane (Fig. 3), and the interspinous distance/intertuberous distance/height of the pelvis ratio:

$$ \begin{array}{cc}\hfill ISD/ITD/HP\hfill & \hfill ratio\hfill \end{array}=\frac{IS{D}_{\left[ mm\right]}}{IT{D}_{\left[ mm\right]}\times H{P}_{\left[ mm\right]}} $$

**Fig. 4:**
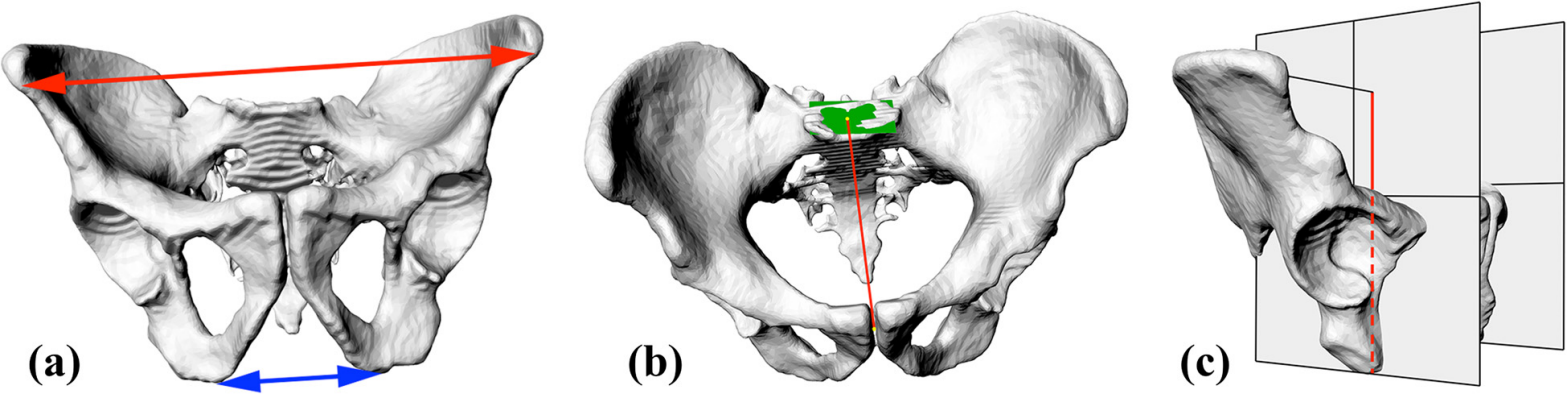
Analysed anthropometric parameters: **a** interspinous distance – marked with the red line; intertuberous distance- blue line; **b** anatomical conjugate – red line; sacrum base plane - green rectangle; **c** Height of the pelvis –red line”

Thus, ISD, ITD, and HP were assessed in each pelvis (Fig. 4).

ISD (Fig. 4) is the distance between the two most prominent points (in the sagittal plane) of the anterosuperior iliac spine (ASIS). ITD (Fig. 4) is the distance between the lowest points (in the sagittal plane) of the ischial tuberosities. HP (Fig. 4) is the relative distance between the ASIS point (projected onto the sagittal plane passing through the ischial tuberosity point) and the ischial tuberosity point (also projected). The iliac opening angle (Fig. 2), separate for left and right pelvic bone, measured based on the cross-section of the pelvis corresponding to the inlet plane, is the angle between the line tangential to the posterolateral wall of the ischial bone and the sagittal axis. The iliac tilt angle (Fig. 4) is the angle formed by the line connecting the ASIS point with the ischial tuberosity center (ITC) and the line between the projection of ASIS on the sagittal plane (as for the HP distance) and the ITC.

**Fig. 5:**
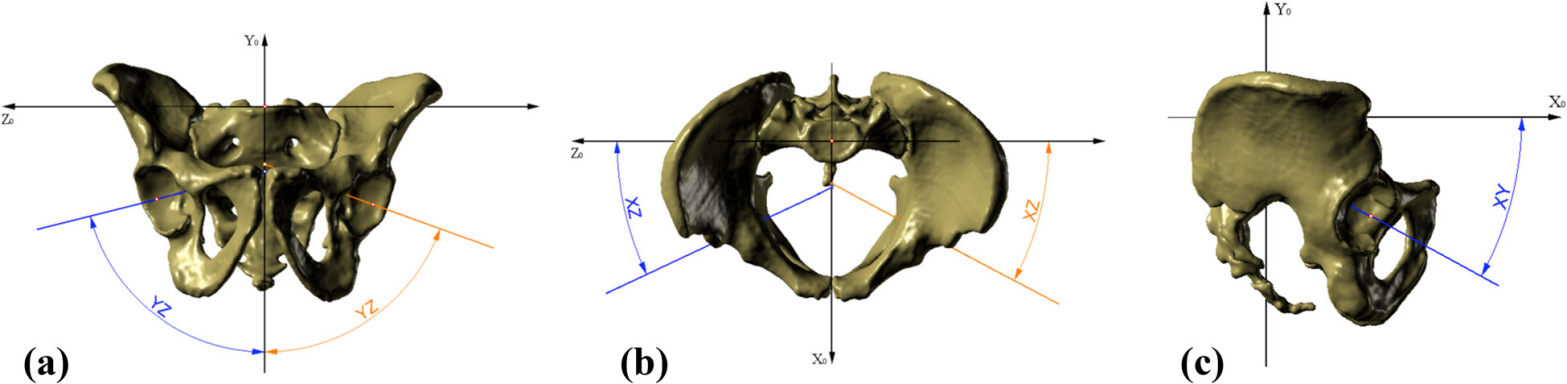
Angles of three-dimensional orientation of the acetabulum. **a** Inclination angle, **b** anteversion angle, and **c** tilt angle

The ISD/ITD/HP ratio was found to describe the span of the iliac wings. The iliac opening angle and iliac tilt angle have not been previously reported in the literature. Based on the axis estimation, the orientation of the acetabulum was described with the use of three angles (Fig. 5): inclination angle, measured in the frontal plane of the pelvis; anteversion angle, measured in the horizontal plane; tilt angle, measured in the sagittal plane [10]. All measurements were obtained with the use of Rhinoceros software.

The linear and nonlinear dependence of the acetabular angles in all planes and hemipelvic measurements were examined with Pearson’s product − moment correlation and Spearman’s rank correlation coefficient. Correlations ≠ 0 with *p* < 0.05 were considered statistically significant. All statistical analyses were performed with the use of STATISTICA software (StatSoft, Tulsa, OK, USA).

The research was a part of the broader research based on CT examinations of human pelvises, which was bioethically approved by the Poznan University of Medical Sciences Bioethical Committee (Ethical approval No. 499/10). All the patients gave general consent for storage and further use of their data in the manuscript.

## Results

Comparison of the axis position (its projection) with the pelvic structure and orientation in the horizontal plane revealed a significant positive correlation between the acetabular anteversion angle and the iliac opening angle, with Pearson’s correlation coefficient and Spearman’s rank correlation coefficient at 0.260 (*p* = 0.041) and 0.239 (*p* = 0.008), respectively (Tables 1, 2).

**Table 1:** Pearson’s correlation coefficients for relations between acetabular angles and pelvic dimensions

Angle	Iliac opening angle	ISD/ITD/HP ratio	Iliac tilt angle
Inclination angle	**−0.359 (** ***p*** **= 0.004)**	**0.253 (** ***p*** **= 0.048)**	**0.285 (** ***p*** **= 0.025)**
Anteversion angle	**0.260 (** ***p*** **= 0.041)**	**0.292 (** ***p*** **= 0.021)**	0.249 (*p* = 0.051)
Tilt angle	**0.628 (** ***p*** **< 0.001)**	0.004 (*p* = 0.978)	0.029 (*p* = 0.823)

*ISD* interspinous distance, *ITD* intertuberous distance, *HP* height of the pelvis

There were 62 cases for each parameter

Statistically significant correlations are in boldface type

**Table 2:** Spearman’s rank correlation coefficient for relations between acetabular angles and pelvic dimensions

	Iliac opening angle	ISD/ITD/HP ratio	Iliac tilt angle
Inclination angle	**−0.248 (** ***p*** **= 0.006)**	**0.253 (** ***p*** **= 0.048)**	**0.312 (** ***p*** **= 0.014)**
Anteversion angle	**0.239 (** ***p*** **= 0.008)**	**0.349 (** ***p*** **= 0.005)**	0.243 (*p* = 0.057)
Tilt angle	**0.613 (** ***p*** **< 0.001)**	0.42 (*p* = 0.748)	0.043 (*p* = 0.741)

*ISD* interspinous distance, *ITD* intertuberous distance, *HP* height of the pelvis

There were 62 cases for each parameter

Statistically significant correlations are shown in boldface type

In the frontal plane, there was a positive correlation between the acetabular inclination angle and the iliac tilt angle, with Pearson’s correlation coefficient and Spearman’s rank correlation coefficient at 0.285 and 0.312, respectively. There was also a positive correlation between the acetabular inclination angle and the ISD/ITD/HP ratio, with Pearson’s correlation coefficient and Spearman’s rank correlation coefficient both at 0.253. There was also a significant positive correlation between the acetabular anteversion angle and the ISD/ITD/HP ratio (Pearson’s coefficient at 0.292 and Spearman’s rank correlation coefficient at 0.349).

In the sagittal plane, there was a positive correlation between the acetabular tilt angle and the iliac opening angle (Pearson’s coefficient at 0.628 and Spearman’s rank correlation coefficient at 0.613) (Tables 1, 2).

**Fig. 6:**
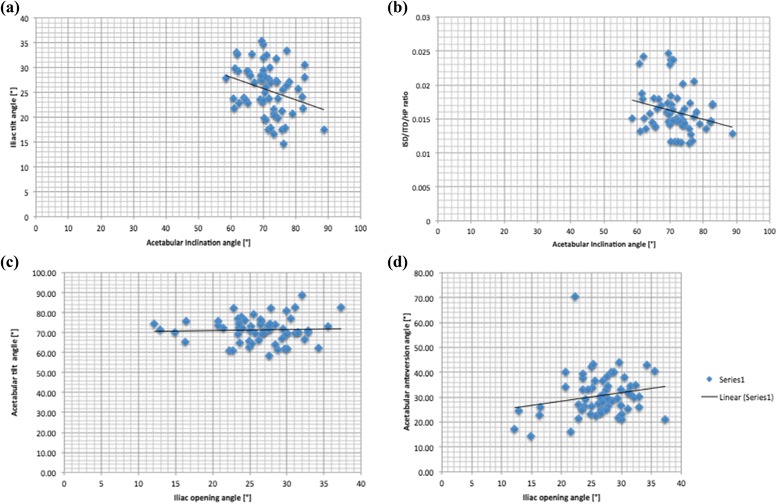
Trend lines of main comparisons: **a** Iliac tilt angle with Acetabular inclination angle; **b** ISD/ITD/HP ratio with Acetabular inclination angle; **c** Iliac opening angle with Acetabular tilt angle; **d** Iliac opening angle with Acetabular anteversion angle

Figure 6 shows trend lines for the main comparisons, including the acetabular inclination angle with the iliac tilt angle and the ISD/ITD/HP ratio as well as the acetabular anteversion and tilt angle with the iliac opening angle. The average values of anthropometric measurements in the pelvis are presented in Table 3, and those of the acetabular angles are in Table 4.

**Table 3:** Average values of pelvic dimensions in examined cases

	ISD (mm)	ITD (mm)	HP (mm)	ISD/ITD/HP ratio	Iliac opening angle (°)	Iliac tilt angle (°)
Average value	251.56	105.91	151.92	0.0161	26.39	25.47
SD	18.32	11.50	16.81	0.0033	5.05	4.97
Min value	210.77	82.14	99.17	0.0114	12.15	14.59
Max value	295.94	124.50	184.95	0.0246	37.32	35.40

*ISD* interspinous distance, *ITD* intertuberous distance, *HP* height of the pelvis, *Min* minimum, *Max* maximum

There were 62 cases for each parameter

**Table 4:** Average acetabular angles in examined cases

	Inclination angle (°)	Anteversion angle (°)	Tilt angle (°)
Average values	71.34	30.15	31.06
SD	6.22	7.25	10.59
Min value	58.52	14.25	13.82
Max value	88.77	43.99	54.91

*Min* minimum, *Max* maximum

There were 62 cases for each parameter

## Discussion

Although the morphologic features of the hip under various physiologic and pathologic conditions have been well described, the structure of the entire pelvis in these hip disorders is not well characterized. The association of the pelvic shape with morphologic features of the acetabulum is even less clear. Only exceptional cases have appeared in the literature on this subject, and they focused on this relation only in developmental hip diseases [14–16]. We found no research that assessed this relation in the presence of secondary hip dysplasia or in adult cases (e.g., FAI). Even more surprising is the fact that there was no research to assess the relation of the acetabulum orientation and pelvic structure under different physiologic conditions. Hence, we focused on measurements of pelvic anthropometric parameters and the acetabular axis and questioned whether there are any significant correlations between them in the frontal, horizontal and sagittal planes.

Our results proved that the acetabular spatial orientation is dependent not only on the pelvic position in space [21–24] but also on its structure per se. To our knowledge, this is the first trial that assessed the relation between the acetabulum and the whole pelvic bone in planes other than the horizontal plane [14]. It is also one of two studies that evaluated the acetabulopelvic relation based on 3D radiographic views and one of the few studies that objectively evaluated the acetabular axis in spatial reconstructions, not plane views [10, 14].

Our results revealed that in the frontal plane, the more tilted-down innominate bone (reflected in an increasing iliac tilt angle) caused a more caudally oriented acetabular axis (increased inclination angle). As a consequence, in patients in whom the pelvic bones are more tilted to the side, there is a greater extent of superior coverage of the femoral head. Fowkes et al. had previously confirmed this observation [25].

Reversed dependence is found in the horizontal plane. There is a direct proportional relation between the acetabulum and the hemipelvis. That is, when the iliac opening angle increases, acetabular anteversion also increases. In other words, with the pelvic bone facing posteriorly, the acetabulum expands its posterior wall, leading to slight movement of its axis anteriorly. An increased anteversion angle could be explained by the need for posterior femoral head coverage, which prevents the hip joint from dislocating, however one must remember that number of other factors, such as pelvic incidence may influence this relation. Another explanation could be a diminishing anterior acetabular wall to prevent dynamic conflict with the femur head [26–28]. These changes in wall size were observed in our material subjectively. Further studies under both physiologic and pathologic (especially in patients with FAI) conditions must be undertaken to confirm this statement.

This study opens the discussion of intrinsic interaction of the pelvic structure and acetabular orientation and architecture (especially in regard to femoral head coverage). Based on the physiologic conditions, studies of acetabulopelvic relations could be evaluated in pathologic situations, especially in cases where the acetabulum is set in retroversion [8, 20, 26, 29–31].

There were several limitations to this study. First, the study included only models of male pelvises to keep it as homogeneous as possible. Further research on female participants could be useful for both unifying the results for the two sexes and for allowing a comparison of the two groups. Second, the tools needed to prepare and subsequently assess the 3D reconstructions of the pelvis on CT were extremely precise, and the assessment itself is time-consuming. In the future, this methodology will have to be modified so the 3D reconstruction software is simpler, although it will probably lose some of its precision. Third, we did not find any good indicator to evaluate the position and structure of the hemipelvis in the sagittal plane. Thus, the relation of acetabular orientation in the sagittal plane (expressed by the acetabular tilt angle) was not directly assessed in this study. Fourth, this study assesses only the intrinsic relations of the pelvis, not taking the external factors, such as pelvic position in relation to coronal plane of human in standing position, under consideration. Some of these factors could influence the internal relations between pelvic bone and acetabulum, however we believe that not significantly enough to change tendencies presented in this research. Nevertheless, further investigations in this subject may be beneficial.

## Conclusions

In conclusion, this study revealed significant correlations of the hemipelvic structure and position with acetabular orientation under anatomic conditions, especially in the frontal and horizontal planes.

## Availability of data and materials

The dataset supporting the conclusions of this article is available upon readers request – please contact corresponding author (ortopediadziecieca@gmail.com).

## Abbreviations

2D: two dimensional
3D: three dimensional
ASIS: anterior superior iliac spine
AVG: average
CAD: computer-aided design
CT: computed tomography
DDH: developmental dysplasia of the hip
FAI: femoroacetabular impingement
ISD: interspinous distance
ITD: intertuberous distance
MAX: maximum
MIN: minimum
N: number of cases
OA: osteoarthritis
SB: the sacrum base
SD: standard deviation
